# Assesment of normal tissue radiosensitivity in cervical tumors

**DOI:** 10.1186/1753-6561-7-S2-P6

**Published:** 2013-04-04

**Authors:** Ana TS Carvalho, Hellen S Cintra, Nathany R Barbosa, Juliana CD Pinezi, Renata BA Soares

**Affiliations:** 1 Laboratory of Oncogenetics and Radiobiology, Goiânia, GO, Brazil; 2 Genetics Master's Program, PROPE, PUC, Goiânia, GO, Brazil; 3 Department of Medicine, PUC, Goiânia, GO, Brazil; 4 Medicine Faculty, Radiology Department, USP, São Paulo, SP, Brazil

## Background

In Brazil, cervical cancer is the second most common malignant tumor among women. Radiation therapy is part of its interdisciplinary management. The major challenge of modern medicine in radiotherapy is to develop predictive methods that can determine the level of radiosensitivity of the patient and the healthy surrounding tissue in order to individualize the prescribed radiation dose, and to prevent severe side effects, promoting better local tumor control. This study evaluated the acute and chronic adverse effects on the skin, lower gastrointestinal tract and urinary tract of radiotherapy in 47 patients with cervical cancer.

## Materials and methods

After signing the informed consent agreement, a sample of peripheral blood of 47 patients was collected then the DNA was extracted. *TP53* and *ATM* sequences were amplified to be sequenced.

## Results

Univariate analysis showed that age was strongly associated with a risk of acute skin toxicity (*p*=0,023). Patients who received a high dose of external beam radiation and patients who have undergone brachytherapy, showed a significantly higher incidence of chronic urinary tract toxicity (*p*=0,031) and (*p*=0,019), respectively. The exchange G>A in the position 5557 of the *ATM* gene was significantly associated with the risk of acute lower gastrointestinal tract toxicity (*p*=0,008).

## Conclusions

Our data corroborate the importance of investigating genetic profiles in order to predict adverse side effects in patients with cervical cancer undergoing radiotherapy. *TP53* and *ATM*, known to play an important role in DNA repair pathways, are probably capable of modifying responses of normal tissues to radiotherapy.

## Financial support

FINEP (Rede GENOPROT) and CNEN.

